# News Coverage of Child Care during COVID-19: Where Are Women and Gender?

**DOI:** 10.1017/S1743923X20000598

**Published:** 2020-08-13

**Authors:** Rebecca Wallace, Elizabeth Goodyear-Grant

**Affiliations:** 1 University of Toronto; 2 Queen's University

**Keywords:** Childcare, child care, gender, women, media framing, social policy, COVID-19, coronavirus, pandemic, Canada, Canadian politics

## Abstract

Research has long observed the absence of gender in child care policy, media, and elections. However, the COVID-19 pandemic has invoked critical questions about child care and its importance to states’ economic recoveries around the world. In this research note, we analyze news coverage of child care in major Canadian daily newspapers to explore whether and how news narratives regarding child care are shifting in light of the COVID-19 pandemic. In particular, are we seeing a focus on women and gender in child care coverage amid the compounding pressures that women face in the current social and economic climate? The results of our analysis suggest that the pandemic has not shifted the conversation on child care and that current coverage principally reflects long-standing trends in child care framing. We find that gender remains systematically written out of coverage of child care, occluded by a larger focus on health-, economic-, and accessibility-related concerns about child care services.

Gender and women do not figure prominently in discussions of child care^1^—not in policy, news coverage, or election campaigns (e.g., Albanese et al. 2010; Collier 2012; Jenson 2009, 2012; Naumann 2012; Wallace and Goodyear-Grant 2020)—despite the issue's centrality to women's success and gender equality. Public debate about child care tends to focus on families and early childhood development, informed by a pro-employment social investment perspective on social policy (e.g., Collier 2012; Jenson 2012; Naumann 2012). Even civil society groups working in the area have moved away from gender or feminist framing (Collier 2012), decentering women—distinct from parents generally—as important clients of the child care system.

The COVID-19 pandemic, however, has created conditions favorable to taking women and gender more seriously in the child care discussion. Globally, schools and child care facilities have been closed or their operations have been significantly reduced for months, starting in mid-March 2020 in Canada. Unsurprisingly, data suggest that women are handling a disproportionate share of the additional work of child care and homeschooling,^2^ widening existing gender gaps in the workplace and the home. At the same time, more men than ever have experienced the difficulties of managing work and child care, whether working inside or outside the home, perhaps sensitizing them to the daily gendered dimensions of working parenthood. Labor force data also reveal that COVID-19-related job losses have been borne disproportionately by women, and economists insist that “there will be no recovery without a she-covery; no she-covery without child care.”^3^

There has been a genuine acknowledgment of the pandemic's disproportionate effects on women, so the question we tackle in this article is whether women and gender have been central to news coverage of child care, which is an essential service for women's resumption of their regular work activities. In this research note, we examine print news coverage in Canada from March 1 to May 31, 2020, to address the question. Overall, we find similar patterns to pre-COVID-19 studies, with very little focus on women or gender (e.g., Albanese et al. 2010; Wallace and Goodyear-Grant 2020). Despite widespread knowledge of the pandemic's gendered effects, news coverage of child care in Canada during the first three months of COVID-19 restrictions has been largely ungendered, with health, economic issues, and accessibility dominating coverage, similar to before the pandemic.

## DATA AND METHODS

To analyze news coverage of child care during the pandemic, we drew a sample of English-language news articles from the Canadian Major Dailies database from March 1 to May 31, 2020.^4^ Articles included in the sample contained a relevant child care keyword in the article headline (“child care,” “childcare,” “daycare*,” “day care*,” “pre-school*,” “preschool*,” or “nursery school*”) combined with “COVID,” “coronavirus,” or “pandemic” anywhere in the text. Our goal was to examine news coverage that focused substantively on the issue of child care, offering an opportunity to closely examine the core themes that have emerged on this critical public policy issue during the pandemic. Moreover, the time frame under study captures the beginning of closures of child care facilities, schools, and nonessential services across the provinces of Canada, as well as the initial stages of reopening in the regions. This allows us to examine the trajectory of discussions about child care over several weeks of the pandemic and to track how different dimensions of the issue shifted or changed in salience over time and in response to coinciding events, outbreaks, or policy changes. After removing duplicate articles, the search yielded a total of 247 news articles from 18 newspapers in Canada.

Using computer-assisted content analysis techniques (see the appendix in the supplementary material for descriptions), we identified four key dimensions of child care in the news sample:

1. Health concerns: Coverage pertaining to health risks in child care facilities and the safety measures that child care facilities have adopted or will adopt to limit viral spread.
2. Economic concerns: Coverage of child care as it relates to the function of the Canadian economy.
3. Accessibility concerns: Coverage related to maintaining child care spaces in light of the pandemic, particularly with the (temporary or permanent) closure of licensed facilities.
4. Gendered concerns: Coverage related to gendered aspects of child care, including the disproportionate burden of care that women have incurred throughout the pandemic.

Next, we describe news coverage of child care over the period, specifically analyzing the frequency of the four dimensions of care in the sample, including any shifts in framing associated with the journalist's sex or the date of coverage.

## RESULTS

**Figure 1.**
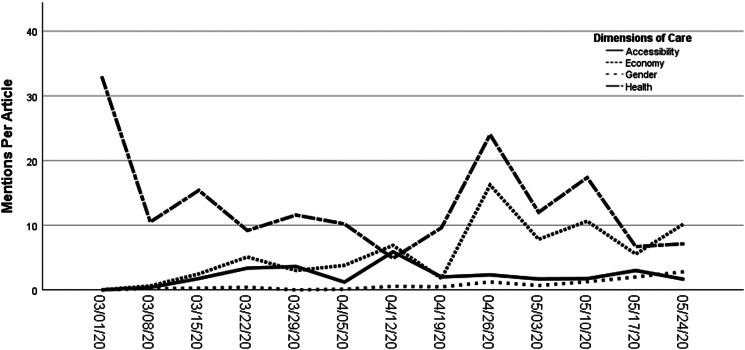
Weekly average mentions of four care dimensions, per article.

Table 1 displays the frequency of frame mentions in the coverage. Perhaps not surprisingly, we find that the most prominent dimension of coverage that emerges during the pandemic is health-related concerns. Descriptions of health risks—and steps that facilities are taking to mitigate them—were raised at least once in 96% of the articles included in the study, suggesting it is a critical component of current debates about the closure and reopening of child care facilities in Canada. Evident in Figure 1, the health dimension was prominent in the early weeks of March amid the closure of care facilities to limit the spread of the virus. The narrative evolved over the ensuing weeks to explore the challenges of operating child care facilities for children of frontline workers and rose again in the final weeks of April and May amid discussions about safety measures in day care facilities to prevent the spread of COVID-19 upon reopening.

**Table 1.** Frequency of frames in coverage

	Mean Mentions Per Article	Maximum Mentions Per Article	Total Mentions in Sample	Std. Deviation of Mentions Per Article
Accessibility	2.18	20	538	3.183
Economy	6.23	54	1540	9.257
Gender	.75	26	174	2.851
Health	13.15	88	3248	13.354

Economic concerns represent the next most prominent theme in coverage of child care during the pandemic. The importance of child care to Canada's economic recovery was raised in 72% of articles in the sample, particularly from the final weeks of April onward (Figure 1, *p* < .001), as several provinces announced their plans for “reopening” the economy. These articles generally discussed the economic fallout from the pandemic and the measures governments have taken to support families and child care businesses to ensure that they are able to reopen in the ensuing phases of the recovery.

Closely linked with the economic dimension are concerns about the accessibility of child care. This dimension refers to the availability of child care, including whether the number of spaces will decline as a result of new distancing and cleaning protocols, as well as how many child care centres will close as a result of economic hardship brought by extended closure or higher operation costs upon reopening. Issues pertaining to the costs and availability of child care—especially amid the closure of many licensed facilities across Canada—were discussed consistently throughout the pandemic thus far, mentioned at least once in 58% of the articles in the sample.

Consistent with previous research in the field, the gender dimension of child care was the least prominent frame in the sample, referenced in only 20% of articles. This means that four out of every five articles on child care did not even mention—let alone carefully analyze—women in the context of the implications of child care policy.

The gender frame most commonly emerges in coverage focused on economic recovery, where often little more than a single mention of women emerges in the context of discussing the caretaking challenges that parents are experiencing during the pandemic. For example, three articles quoted Conservative Party leadership candidate Erin O'Toole, stating, “Far more women than men have lost their jobs, a reflection of how hard the hospitality, service and retail sectors have been hit . . . With schools and daycares closed, many workers with kids—particularly women—wonder how they will be able to get back to work” (Platt 2020). This notion that women are “especially” or “particularly” hard hit by the current crisis as mentioned in 14% of the articles in the sample, which did not further explore women's experiences with balancing care and work responsibilities or how gendered care imbalances affect women's economic success, personal well-being, or, indeed, the COVID-19 economic recovery itself.

Only 6% of the articles in the sample substantively discussed gendered-related issues regarding care work, referring to women or gender more than twice in an article. These articles highlighted the challenges that many women have faced in balancing their time between paid work and caregiving work, as well as the implications this could have on women's productivity and career advancement in the future. As one article described, “time away from work might mean not getting promotions or building up work hours associated with career advancement. As well, staying home means not paying into a pension plan or employment insurance, including maternity and paternity leave” (Taylor 2020).

The coverage of child care during the pandemic also remains silent on a multitude of gender-based inequalities—social and economic—that mothers face in taking on a larger proportion of child care work. For example, only 2% of articles in the sample acknowledged issues related to equal pay and the wage gap. Many women also face additional demands in terms of caring for seniors, which were mentioned in 1% of the articles. Further still, discussions of single mothers remain absent from the conversation on child care, similarly featured in 1% of the articles in the sample. Domestic violence, which has been on the rise since the beginning of quarantine in Canada (Illesinghe 2020) and is integrally linked to familial care, is mentioned in fewer than 4% of articles. In effect, it seems the gendered issues related to child care during the pandemic have most frequently been conceptualized in relation to Canada's economic recovery, with little substantive focus on the diverse experiences, challenges, and barriers that mothers continue to face.

As Figure 1 reflects, while the other child care frames show shifts in frequencies over the timeline, gender framing did not see any marked variation throughout the time period under study. Gender and women, it seems, were background, low-salience features of public discourse on child care during the early stages of the pandemic shutdown and early phases of “reopening.” Moreover, consistent with our previous research on the framing of child benefits (Wallace and Goodyear-Grant 2020), we also find that women journalists are more inclined than their male colleagues to speak about gender in coverage of child care (*p* < .001). Although there is relative parity in stories written by male and female journalists,^5^ women journalists account for 80% of the gendered mentions in the sample, while male journalists account for a mere 20%. While this may be unintentional—and perhaps even a product of editorial teams’ decisions to have women journalists write about women's issues—it suggests that we need to further explore the ways that women's representation may matter when it comes to ensuring that gendered perspectives are on the table in child care policy debates during COVID-19 recovery efforts.

## CONCLUSIONS

Around the world, child care policy and practice have critical gender-related dimensions. Accessible, affordable child care is a necessary ingredient for women's equality, especially in the context of economic and social recovery from COVID-19. Our analyses suggest that the media information environment has not engaged sufficiently with these realities and may be contributing to the neglect of women and gender in this policy area, the normalization of gender-related care imbalances, and the removal of these challenges from the public eye and public accountability.

## Footnotes

1. We analyze coverage of care arrangements for preschool-aged children. Of course, child care or care for children encompasses more than this and includes school-aged children, especially during COVID-19, when children were not in school in most jurisdictions and were doing or attempting remote schooling in many cases. Our analyses only relate to coverage of child care for preschool-aged children.

2. See, e.g., Maya Oppenheim, “Coronavirus: Women Bearing Burden of Childcare and Homeschooling in Lockdown, Study Finds,” *The Independent*, May 14, 2020, https://www.independent.co.uk/news/uk/home-news/coronavirus-childcare-homeschooling-women-lockdown-gender-a9512866.html; Claire Cain Miller, “Nearly Half of Men Say They Do Most of the Home Schooling. 3 Percent of Women Agree,” *New York Times*, May 6, 2020, https://www.nytimes.com/2020/05/06/upshot/pandemic-chores-homeschooling-gender.html (both accessed August 24, 2020).

3. Economist Armine Yalnizyan's statement to the House of Commons Standing Committee on Human Resources, Skills and Social Development and the Status of Persons with Disabilities, June 4, 2020, https://atkinsonfoundation.ca/atkinson-fellows/posts/no-she-covery-without-child-care/ (accessed August 24, 2020).

4. This analysis excludes French-language news articles, as the dictionaries have not been translated and validated in French to date. While the analysis still includes some English news sources from Quebec, such as the *Montreal Gazette,* the exclusion of French media may overlook important debates pertaining to gender and childcare in the province. Quebec maintains its own family and child policy supports that are separate from or in some cases additional to national programs/services, and media coverage of childcare during the pandemic may raise different perspectives on existing supports, challenges, or policy gaps. We acknowledge that this is an important limitation of the analysis, and future iterations of this work intend to adopt a more comparative framework for analyzing the framing of child care between Quebec and the rest of Canada in this uncertain time.

5. Women journalists wrote 90 articles in the sample (53% of stories included in the journalist gender analyses), while male journalists wrote 80 (47%).

## Supplementary material

For supplementary material accompanying this paper visit http://dx.doi.org/10.1017/S1743923X20000598. click here to view supplementary material
