# Supplementary material for: News Coverage of Child Care during COVID-19: Where Are Women and Gender?
Source: Politics & Gender. 2020 Aug 13:1–8. doi: 10.1017/S1743923X20000598 (PMC7562856; doi:10.1017/S1743923X20000598)
Supplement: Supplementary file 1 [file S1743923X20000598sup001.docx]

**Appendix**

**Description of Automated Text Analysis**

Data analyses were conducted using WordStat, an automated text analysis program that allowed us to examine key frames in the text. Consistent with common practices in the field of automated text analysis (see Wallace 2018; Wallace and Goodyear-Grant 2020), we began by extracting the most common words and phrases utilized in the news sample and, using WordStat’s dendrogram function, explored the co-occurrences of such terms across the articles under study. This function of the program uses Jaccard’s coefficient to determine the co-occurrence of keywords in each article in the sample, which highlights the core underlying themes in the corpus. We identified four core themes pertaining to child care in the coverage, and further fleshed out the four dictionaries by adding related terms, as well as inclusion or exclusion rules to ensure only the intended use of keywords were retrieved by the dictionaries. This meant, for example, in the economic dictionary that we only included references to recovery (“recover*”) that mentioned the economy (“econom*”) in the same sentence, to ensure that the dictionary did not count references to patients recovering from the virus.

Although automated coding is generally very efficient and effective, it is still important to manually validate results. As such, we checked each dictionary term using the Keyword in Context function and looked at 20% of paragraphs where the term was mentioned to ensure that the context, interpretation, and meaning were correct. Similarly, following from common practices in the field of automated content analysis (e.g., Lawlor and Tolley 2017; Wallace 2018; Wallace and Goodyear-Grant 2020), we analyzed a random sample of 20% of the articles in the corpus and conducted a manual check to ensure that the coding of the terms in our dictionaries were correct. Errors were corrected and capture-specific rules applied throughout this iterative process until there was at least 90% agreement between automated and manual coding results.

In terms of independent variables, we analyzed the trends in coverage over the timeline and in accordance with the journalists’ gender. The researchers determined the journalists’ gender by searching for journalists’ biographies or portfolios online, deriving their gender from their chosen pronouns. Articles that did not list authors, or where the authors’ biographies could not be found, were excluded from this portion of the analysis. Articles that were cowritten by multiple journalists that included at least one female journalist were categorized under women journalists. While this is not a perfect measure that accounts for the diversity of sex and gender—and the ways that it could affect one’s positionality in relation to child care and family policy—our goal here was to assess if journalists who identified as women offered a different account of child care than their men colleagues. We view this as a starting point for future analyses that dig deeper into these more complex questions about critical actors and advocacy work in the area of child care policy.

**References**

Lawlor, Andrea, and Erin Tolley. 2017. “Deciding Who’s Legitimate: News Media Framing of Immigrants and Refugees.” *International Journal of Communication* 11: 25.

Wallace, Rebecca. 2018. “Contextualizing the Crisis: The Framing of Syrian Refugees in Canadian Print Media.” *Canadian Journal of Political Science/Revue canadienne de science politique* 51 (2): 207–31.

Wallace, Rebecca, and Elizabeth Goodyear-Grant. 2020. “Writing Gender Out or Working It Back In? Media Coverage of Child Benefits in Canada.” *Journal of Women*, *Politics & Policy*. Published online August 5. <https://doi.org/10.1080/1554477X.2020.1783932>.
